# Cranial Giant Cell Arteritis Presenting as Pyrexia of Unknown Origin: A Diagnostic Challenge With Negative PET-CT but a Positive Halo Sign

**DOI:** 10.7759/cureus.113811

**Published:** 2026-08-02

**Authors:** Alan Rachid, Ameer A Khan, Nevin Omer, Honar Rachid, Mohammed Fadgham, Hisham Hago Bannaga Hassan, Amber Khan

**Affiliations:** 1 General Internal Medicine, Tameside General Hospital, Ashton-Under-Lyne, GBR; 2 Cardiology, Tameside General Hospital, Ashton-Under-Lyne, GBR; 3 General Dentistry, Eskişehir Osmangazi University, Eskişehir, TUR; 4 General Internal Medicine, Marienhospital, Aachen, DEU; 5 General Internal Medicine, Salford Royal Hospital, Salford, GBR

**Keywords:** atypical presentation, giant-cell arteritis, puo (pyrexia of unknown origin), temporal arteritis (ta), temporal artery ultrasound (taus)

## Abstract

Giant cell arteritis (GCA) classically presents with cranial symptoms, but systemic features may predominate and obscure the diagnosis. We report the case of a woman in her early 70s who presented with a six-week history of nausea, vomiting, anorexia, weight loss, and intermittent confusion, followed by persistent pyrexia during a prolonged inpatient admission. Extensive investigations for infection, malignancy, and systemic autoimmune disease were unrevealing, including computed tomography (CT) imaging, echocardiography, microbiological testing, autoimmune panels, and fluorodeoxyglucose positron emission tomography/computed tomography (FDG-PET/CT). She received five courses of broad-spectrum intravenous antimicrobial therapy without a clinical or biochemical response. Her C-reactive protein remained above 120 mg/L for several weeks. Despite the absence of new headache, jaw claudication, scalp tenderness, or visual symptoms, temporal artery ultrasound was requested because of concern for atypical GCA. This demonstrated bilateral incompressible echolucent halos, consistent with cranial GCA. Prednisolone 40 mg daily was commenced, after which the fever resolved within 48 hours, cognition normalised, and oral intake improved substantially. She was discharged after 51 days on a tapering corticosteroid regimen. This case emphasises that GCA should remain in the differential diagnosis of pyrexia of unknown origin in older adults with sustained systemic inflammation and a poor response to antimicrobial therapy, even when classical cranial symptoms are absent and FDG-PET/CT does not demonstrate large-vessel vasculitis. Early temporal artery ultrasound may reduce diagnostic delay, unnecessary antimicrobial exposure, and prolonged hospitalisation.

## Introduction

Giant cell arteritis (GCA) is the most common primary systemic vasculitis affecting older adults and most frequently involves branches of the external carotid artery [1]. The classical presentation includes new-onset headache, scalp tenderness, jaw or tongue claudication, and visual disturbance, with polymyalgia rheumatica symptoms coexisting in up to 50% of cases [2]. Systemic manifestations, including fever, weight loss, malaise, and anaemia, are also recognised, although they more commonly accompany rather than replace cranial features.

Pyrexia of unknown origin (PUO), defined as fever exceeding 38.3°C for more than three weeks without a diagnosis after appropriate inpatient investigation, is a recognised but atypical presentation of GCA [3]. When fever is the dominant or sole manifestation and when headache, jaw claudication, and visual symptoms are absent, the diagnosis may be delayed as patients undergo repeated antimicrobial treatment and extensive investigations [4].

Sverdlichenko et al. conducted a review that found that 18% of patients with biopsy-proven GCA and atypical features had no concurrent headache, jaw claudication, or visual symptoms. Treatment initiation was substantially delayed in this atypical-only group compared with patients who had typical features (median 450 days versus 3 days, p < 0.001) [5].

We describe a case of ultrasound-confirmed cranial GCA in a woman in her early 70s whose presentation was dominated by PUO, confusion, vomiting, and weight loss. The diagnosis was established by temporal artery ultrasound and was followed by a rapid clinical response to corticosteroid treatment. This case reinforces the importance of considering GCA in older adults with unexplained fever and persistent inflammation and highlights the diagnostic value of temporal artery ultrasound in this setting [6,7].

## Case presentation

A woman in her early 70s was admitted to the medical unit with a six-week history of worsening malaise, loss of appetite, nausea, and intractable vomiting. She had been unable to tolerate solid food or oral medications for two weeks before admission and had experienced significant unintentional weight loss. Her family also reported intermittent confusion over the preceding days. She denied new headache, visual disturbance, jaw pain, scalp tenderness, and limb claudication. Her past medical history included type 2 diabetes mellitus, hypertension, hypercholesterolaemia, depression, asthma, and vitamin D deficiency. Her regular medications included metformin, atorvastatin, ramipril, duloxetine, bezafibrate, bisoprolol, lercanidipine, canagliflozin, darifenacin, aspirin, and omeprazole.

On admission, she was afebrile with a NEWS2 (National Early Warning Score) of 0 but appeared frail and cachectic. Cardiovascular, respiratory, and abdominal examinations were unremarkable. There was no temporal artery tenderness or peripheral oedema. Neurological examination showed no focal deficit, although she was intermittently confused and showed signs of paranoia, specifically concerns that staff were interfering with her cannulas.

Investigations

Initial blood tests showed a markedly elevated C-reactive protein (CRP) level of 280 mg/L, a white cell count of 24.9 × 10^9^/L with a neutrophil count of 21.3 × 10^9^/L, and normocytic anaemia with a haemoglobin level of 115 g/L. Liver function tests showed a raised alkaline phosphatase level of 145 U/L. Procalcitonin was only mildly elevated at 0.13 µg/L. Autoimmune, vasculitis, and connective tissue disease screens were also negative. Table 1 summarises the blood tests performed during the initial investigations. 

**Table 1:** Blood tests as part of initial investigations RBC: red blood cell, MCV: mean corpuscular volume, MCH: mean corpuscular hemoglobin, MCHC: mean corpuscular hemoglobin concentration, RDW: red cell distribution width, WBC: white blood cell count, ESR: erythrocyte sedimentation rate, LA: lupus anticoagulant, NPP: normalized plasma pool, APTT: activated partial thromboplastin time, ANCA: antineutrophil cytoplasmic antibody, PR3: proteinase 3, MPO: myeloperoxidase, AI: antibody index, IgG: immunoglobulin G, IgA: immunoglobulin A, IgM: immunoglobulin M, CMV: cytomegalovirus, EBV: Epstein-Barr virus, VCA: viral capsid antigen, ALT: alanine aminotransferase, eGFR: estimated glomerular filtration rate, TSH: thyroid-stimulating hormone, T4: thyroxine, NT-proBNP: N-terminal pro-B-type natriuretic peptide.

Blood tests	Result	Reference range
Haemoglobin	~86 to 79 to 93 g/L during the early admission period; possible 61 g/L later during admission, subsequently improving to 93 g/L	115-165 g/L
RBC count	~3.50, 3.47, then 2.58 to 3.47 × 10¹²/L	3.8-5.8 × 10¹²/L
Haematocrit	~0.26-0.30 L/L	0.36-0.46 L/L
MCV	~74-76 fL	80-100 fL
MCH	~22.8-24.0 pg	27-32 pg
MCHC	318 to 305 g/L during admission	320-360 g/L
RDW	16.1 to 17.0%	11.5-14.5%
WBC	10.0 to ~11.8 × 10⁹/L during admission	4.0-11.0 × 10⁹/L
Neutrophils	7.3 to 9.6 × 10⁹/L	1.8-7.5 × 10⁹/L
Lymphocytes	1.9 to 1.4 × 10⁹/L	1.0-4.0 × 10⁹/L
Monocytes	0.7 to 0.6 × 10⁹/L	0.2-0.8 × 10⁹/L
Eosinophils	0.1 × 10⁹/L	0.0-0.4 × 10⁹/L
Basophils	0.0 to 0.1 × 10⁹/L	0.0-0.1 × 10⁹/L
Platelets	206 to 286 × 10⁹/L	150-450 × 10⁹/L
ESR	68 mm/hour early during admission; 57 mm/hour later during admission	1-20 mm/hour
Lupus anticoagulant interpretation	Not detected	Not detected
LA screen/NPP	1.55	0.80-1.20
APTT, LA-sensitive	31.3 seconds	24-35 seconds
LA confirm/NPP	1.30	0.80-1.20
Screen/confirm ratio	1.19	<1.20
Standard APTT	26.5 seconds	23-32 seconds
APTT ratio	~0.91-0.93	0.8-1.2
Rheumatoid factor	<10.0	0.0-13.0 kU/L
ANCA fluorescence	Negative	Negative
PR3 antibody	<0.2	0.0-0.9 AI
MPO antibody	<0.2	0.0-0.9 AI
Anti-centromere antibody	<0.2	0.0-0.9 AI
Bioplex connective-tissue disease screen	Negative	Negative
Anticardiolipin IgG	<1.6	<10 GPL U/mL
Anticardiolipin IgM	<1.3	<10 MPL U/mL
C3	1.94, later 2.15 g/L	0.75-1.65 g/L
C4	0.67, later 0.61 g/L	0.14-0.54 g/L
Total IgG	~4.29 g/L	6.0-16.0 g/L
IgA	1.59 g/L	0.8-4.0 g/L
IgM	~0.48 g/L	0.5-2.0 g/L
IgG subclass 1	3.101 g/L	3.82-9.29 g/L
IgG subclass 2	1.327 g/L	2.42-7.00 g/L
IgG subclass 3	0.207 g/L	0.22-1.76 g/L
IgG subclass 4	0.103 g/L	0.04-0.86 g/L
Serum immunofixation	No abnormality detected	No monoclonal protein detected
CMV IgG	Not detected	Not detected
CMV IgM	Not detected	Not detected
EBV VCA IgG	Positive	Not detected
EBV VCA IgM	Negative	Not detected
Hepatitis B surface antigen	Not detected	Not detected
Hepatitis B core antibody	Not detected	Not detected
Sodium	151 mmol/L; another value ~147 mmol/L	133-146 mmol/L
Potassium	3.0 mmol/L	3.5-5.3 mmol/L
Chloride	Not reliably legible	95-108 mmol/L
ALT	25 U/L	7-35 U/L
Magnesium	0.71 mmol/L	0.70-1.00 mmol/L
Creatine kinase	34 U/L; later ~60-70 U/L	25-200 U/L
Ferritin	173 µg/L	15-150 µg/L
eGFR	~84 mL/minute/1.73 m²	≥90 mL/minute/1.73 m²
High-sensitivity troponin I	N/A	<16 ng/L (female 99th centile)
Total cholesterol	~6.70 mmol/L	<5.0 mmol/L (desirable)
TSH	~1.2 mU/L	0.35-5.50 mU/L
Free T4	~12 pmol/L	10.0-20.0 pmol/L
Serum cortisol	~834 nmol/L	145-619 nmol/L
Vitamin D	~72 nmol/L	50-200 nmol/L
NT-proBNP	~127 ng/L	<400 ng/L

Chest radiography, CT brain, CT abdomen and pelvis, and CT thorax did not identify a source of infection, malignancy, or other acute pathology. Blood cultures, urine cultures, and respiratory viral testing were negative. Echocardiography did not demonstrate valvular vegetation.

Over the following two weeks, the patient remained persistently pyrexial, with temperatures reaching 38.4°C on most evenings, despite consistent broad-spectrum intravenous antimicrobials, including gentamicin (days 1-2), co-amoxiclav (days 1-6), piperacillin-tazobactam (days 6-11), clarithromycin (days 8-13), and meropenem (days 11-25). Her CRP remained elevated between 125 and 158 mg/L, and her anaemia progressed, with her haemoglobin dropping to 61 g/L, requiring transfusion. Oesophagogastroduodenoscopy was normal. MRI brain showed disproportionately enlarged lateral ventricles and Sylvian fissures, raising the possibility of normal pressure hydrocephalus; however, subsequent neurology review considered this unlikely to explain the patient’s acute presentation. CT aortography was also performed and showed no evidence of large-vessel vasculitis.

As a final investigation, an FDG-PET/CT was performed during the admission; however, it did not demonstrate a metabolically active focus of infection or vasculitis [8,9].

Diagnosis, treatment, and outcome

**Figure 1:**
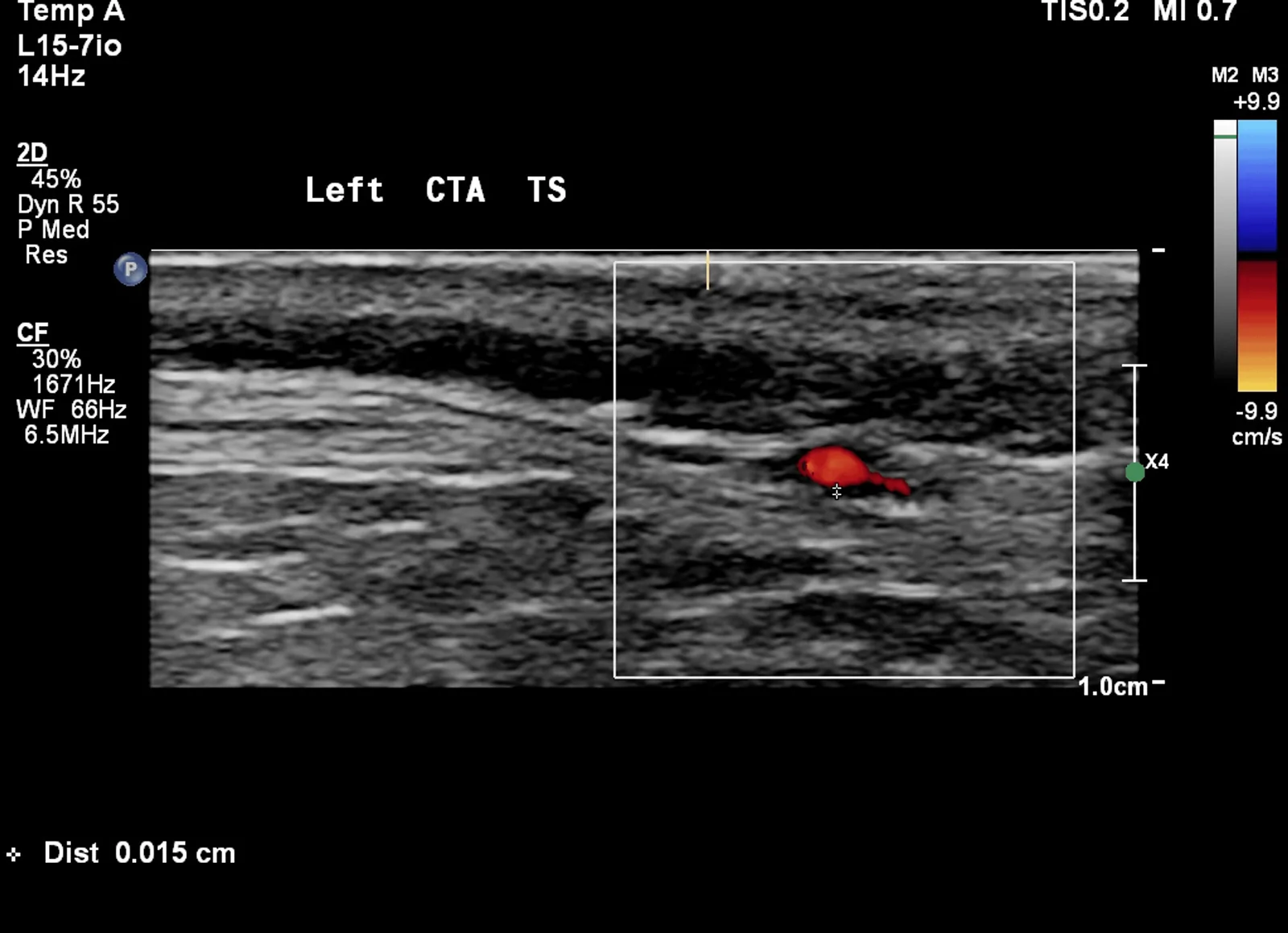
Halo sign demonstrated on temporal artery ultrasound in red

Given the patient's age, persistent fever, markedly elevated inflammatory markers, progressive anaemia, and absence of an identifiable infective source, a consultant requested temporal artery ultrasound to assess for atypical GCA because of the diagnostic conundrum. Ultrasound demonstrated incompressible echolucent halos in the frontal and parietal branches bilaterally, with intima-media thickness measuring up to 0.7 mm in the left parietal branch. The radiology report concluded that the appearances were highly suspicious for right-sided GCA and positive for left-sided GCA. The temporal artery ultrasound halo sign has a reported sensitivity of 76% and specificity of 93% for GCA [10,11]. Figure 1 shows the temporal artery ultrasound demonstrating the halo sign. 

Prednisolone 40 mg daily was commenced. Within 48 hours, the patient became apyrexial for the first time in more than six weeks. Her confusion resolved, vomiting ceased, and oral intake improved substantially. Inflammatory markers improved, with her ESR falling from 41 to 5 mm/hour over the subsequent two weeks. She was discharged home on day 51 on a tapering prednisolone regimen with a proton pump inhibitor and bone protection. Follow-up was arranged with the rheumatology team, and current records indicate no relapse requiring admission.

A diagnosis of GCA with an atypical, fever-dominant presentation was recorded at discharge. A timeline of key events is presented in Table 2.

**Table 2:** Summary of clinical timeline CRP: C-reactive protein, MRI: magnetic resonance imaging, GCA: giant cell arteritis.

Day	Event
0	Admission: 6-week history of nausea, vomiting, poor oral intake and weight loss
1-14	Broad-spectrum antimicrobials; persistent fever; negative cultures and imaging
15-30	Multiple antimicrobial changes; CRP 125-158 mg/L; haemoglobin 61 g/L; MRI brain suggests possible normal pressure hydrocephalus
32	Temporal artery ultrasound demonstrates bilateral features of GCA
33	Prednisolone 40 mg daily commenced
35	Apyrexial; confusion resolved; eating well
51	Discharged on tapering corticosteroids

## Discussion

This case illustrates how cranial GCA can masquerade as PUO with neurocognitive and gastrointestinal features, leading to prolonged antimicrobial exposure and extensive investigation when classical cranial symptoms are absent. The diagnosis was ultimately prompted by the combination of age over 50 years, sustained inflammation, progressive anaemia, weight loss, and failure to identify an infective or malignant source.

Fever as a manifestation of GCA is well described but may be overlooked in routine clinical practice. In a prospective cohort of patients with biopsy-proven GCA, 42% had fever at diagnosis, and fever was the sole presenting feature in 15% [3]. The systematic review by Sverdlichenko et al. is particularly relevant: among 746 patients with atypical GCA features, 18% had only atypical features, without headache, jaw claudication, or visual symptoms, and experienced markedly longer delays before treatment [5]. Our patient fits this atypical-only phenotype. The mechanism of fever in GCA is thought to be cytokine-mediated, involving interleukin-1, interleukin-6, and tumour necrosis factor-alpha released from inflamed vessel walls, rather than a direct consequence of cranial ischaemia [12].

Several factors contributed to the diagnostic delay. First, the absence of new headache, jaw claudication, scalp tenderness, and visual disturbance reduced the perceived likelihood of GCA, despite the patient's age and inflammatory profile [4]. Although her family reported a longstanding, dull, intermittent headache, this was considered baseline and was not consistent with a new acute cranial syndrome. Second, negative microbiological testing, autoimmune screening, and CT aortography did not provide an alternative diagnosis but may have reduced the momentum towards targeted cranial vascular imaging. Third, FDG-PET/CT did not demonstrate vasculitis. FDG-PET/CT remains useful when large-vessel involvement is suspected and has been used in atypical GCA presentations, but it has recognised limitations in cranial-limited disease, particularly when inflammation is confined to the temporal artery branches [9,13,14]. A negative FDG-PET/CT should therefore not exclude GCA when clinical suspicion persists.

Temporal artery ultrasound was decisive in this case. The bilateral halo sign provided a rapid, non-invasive, and highly specific diagnostic clue, allowing corticosteroid treatment to be initiated without waiting for temporal artery biopsy. Diagnostic performance has improved substantially: a meta-analysis reported a pooled sensitivity of 76% and specificity of 93% for the halo sign compared with temporal artery biopsy, and a Cochrane review confirmed the high specificity of the ultrasound halo sign [10,11,15].

Our case highlights the importance of applying the revised EULAR (European Alliance of Associations for Rheumatology) criteria in clinical practice, particularly in atypical presentations such as pyrexia of unknown origin. It also highlights the growing role of non-invasive imaging techniques as first-line diagnostic tools in GCA. Table 2 presents the EULAR classification criteria for giant cell arteritis. 

**Table 3:** 2022 American College of Rheumatology and EULAR classification criteria for giant cell arteritis ESR: erythrocyte sedimentation rate, CRP: C-reactive protein, US: ultrasound, FDG-PET: fluorodeoxyglucose positron emission tomography, GCA: giant cell arteritis, EULAR: European Alliance of Associations for Rheumatology.

Absolute requirement	
Age ≥ 50 years at the time of diagnosis	
Additional clinical criteria	
Morning stiffness in neck /shoulders	+2
Sudden visual loss	+3
Jaw or tongue claudication	+2
New temporal headache	+2
Scalp tenderness	+2
Abnormal examination of the temporal artery	+2
Laboratory, imaging, and biopsy criteria	
Maximum ESR ≥ 50mm/hour or CRP ≥ 10 mg/l	+3
Positive temporal artery biopsy or halo sign on temporal artery US	+5
Bilateral axillary involvement	+2
FDG-PET activity throughout the aorta	+2

The response to corticosteroids was both therapeutic and diagnostically supportive. The patient became apyrexial within 48 hours, and her symptoms improved rapidly. This dramatic response is consistent with reported steroid responsiveness in GCA [2]. The resolution of cognitive symptoms is also consistent with descriptions of potentially reversible cognitive impairment in atypical GCA [16].

While temporal artery biopsy was not performed in this case, it is important to note that a positive ultrasound scan of the temporal and axillary arteries can be used as a confirmatory diagnostic tool for giant cell arteritis. This is reinforced by the British Society for Rheumatology’s updated guidelines on temporal artery biopsy [17]. The evidence shows the importance of obtaining an early ultrasound scan, as ultrasound findings, namely the halo sign, may not be visible if glucocorticoid therapy has already been initiated [17]. It is also worth noting that whilst either temporal artery biopsy or ultrasound of the relevant vessels may be used, there is strong evidence to suggest that performing both tests, where possible, would provide higher diagnostic accuracy in detecting giant cell arteritis [17].

Limitations

This case has several limitations. Temporal artery biopsy was not performed when GCA was first suspected, and treatment was commenced without histological confirmation, leaving the diagnosis dependent on the ultrasound findings and a rapid but non-specific corticosteroid response. It is also important to note that this is a single case with limited long-term follow-up, so subsequent relapse, vascular complications, and sustained treatment response could not be assessed.

## Conclusions

GCA should be considered in older adults with PUO, a marked inflammatory response, anaemia, weight loss, and a poor response to antimicrobial therapy, even when headache, jaw claudication, scalp tenderness, and visual symptoms are absent. In this case, temporal artery ultrasound provided a rapid and non-invasive route to diagnosis after extensive investigations, and corticosteroid treatment produced clinical improvement within 48 hours. The case also demonstrates the limitations of relying on FDG-PET/CT to exclude cranial-limited GCA: a negative scan should not deter targeted vascular ultrasound when clinical suspicion persists. Earlier recognition of GCA may reduce unnecessary antimicrobial exposure and help avoid prolonged hospitalisation.
